# Author Correction: Maladaptive positive feedback production of ChREBPβ underlies glucotoxic β-cell failure

**DOI:** 10.1038/s41467-022-33243-7

**Published:** 2022-09-27

**Authors:** Liora S. Katz, Gabriel Brill, Pili Zhang, Anil Kumar, Sharon Baumel-Alterzon, Lee B. Honig, Nicolás Gómez-Banoy, Esra Karakose, Marius Tanase, Ludivine Doridot, Alexandra Alvarsson, Bennett Davenport, Peng Wang, Luca Lambertini, Sarah A. Stanley, Dirk Homann, Andrew F. Stewart, James C. Lo, Mark A. Herman, Adolfo Garcia-Ocaña, Donald K. Scott

**Affiliations:** 1 Diabetes, Obesity and Metabolism Institute, Icahn School of Medicine at Mount Sinai, One Gustave L. Levy Place, Box 1152, New York, 10029 USA; 2 Pharmacologic Sciences Department, Stony Brook University, Stony Brook, NY USA; 3 Metabolic Phenotyping Core, University of Utah, 15N 2030 E, 585, Radiobiology building, Room 151, Salt Lake City, UT 84112 USA; 4 Weill Center for Metabolic Health and Division of Cardiology, Department of Medicine, Weill Cornell Medicine, New York, NY 10021 USA; 5 Institut Cochin, Université de Paris, INSERM, CNRS, F-75014 Paris, France; 6 Alpenglow Biosciences, Inc., 98103 Seattle, WA USA; 7 12800 East 19th Ave, Anschutz Medical Campus, Room P18-9403, University of Colorado, Aurora, CO 80045 USA; 8 Division of Endocrinology and Metabolism and Duke Molecular Physiology Institute, Duke University Medical Center, Durham, NC USA; 9 Section of Diabetes, Endocrinology, and Metabolism, Baylor College of Medicine, One Baylor Plaza, MS: 185, R614, 77030 Houston, TX USA

**Keywords:** Carbohydrates, Diabetes, Mechanisms of disease, Nuclear transport

Correction to: *Nature Communications* 10.1038/s41467-022-32162-x, published online 30 July 2022

The original version of the Supplementary Information associated with this Article was missing the Supplementary figures. In addition, the legend of Figure 6 contains two errors, where the supplementary figures were incorrectly referenced. It read: “LSL-ChREBPβ mice were bred with MIP-Cre-ERT mice to generate inducible β-cell-specific overexpressing ChREBPβ mice, termed iβOEβ (created with BioRender.com; see also Supplementary Fig. 9). Presented are measurements from male mice (see also Supplementary Fig. 10 for female mice). It should read “LSL-ChREBPβ mice were bred with MIP-Cre-ERT mice to generate inducible β-cell-specific overexpressing ChREBPβ mice, termed iβOEβ (created with BioRender.com; see also Supplementary Figs. 16 and 17). Presented are measurements from male mice (see also Supplementary Fig. 18 for female mice)”.

These errors have been corrected in the PDF and HTML version of the article, and the HTML has been updated to include a corrected version of the Supplementary Information.

## Supplementary information

Updated Supplementary Information

