# Supplementary material for: Maladaptive positive feedback production of ChREBPβ underlies glucotoxic β-cell failure
Source: Nat Commun. 2022 Jul 30;13:4423. doi: 10.1038/s41467-022-32162-x (PMC9339008; doi:10.1038/s41467-022-32162-x)
Supplement: Supplementary file 1 — Supplementary Information [file 41467_2022_32162_MOESM1_ESM.pdf]

Supplementary Materials:

Maladaptive Positive Feedback Production of ChREBPβ Underlies Glucotoxic β-Cell Failure

Supplementary Table 1.

Human primers

Gene	Froward primer	Reverse primer
ChREBPα	ACTCGGACTCGGACACAGAC	AGGCTCAAGCACTCGAAGAG
ChREBPβ	CTGCAGGTCGAGCGGATT	GTCTGTGTCCGAGTCCGAGT
Pklr	CTGGTGATTGTGGTGACAGG	TGGGCTGGAGAACGTAGACT
Txnip	TGTGTGAAGTTACTCGTGTCAAA	GCAGGTACTCCGAAGTCTGT
β-actin	GTCTTCCCCTCCATCGTG	AGGTGTGGTGCCAGATTTTC

Mouse primers

Gene	Froward primer	Reverse primer
ChREBPα	CGACACTCACCCACCTCTTC	TTGTTCAGCCGGATCTTGTC
ChREBPβ	TCTGCAGATCGCGTGGAG	CTTGTCCCGGCATAGCAAC
INS-1	TATAAAGCTGGTGGGCATCC	GGGACCACAAAGATGCTGT
Ins2	TTTGTCAAGCAGCACCTTT	AGG TTT TCT CGC CCC TTA AC
Pklr	TCAAGGCAGGGATGAACATTG	CACGGGTCTGTAGCTGAGTG
Txnip	CTCGGGTGGAGTGCTTAGAG	CTGATGGAGGCACAGTGAGA
ACC	CGCTCACCAACAGTAAGGTGG	GCTTGGCAGGGAGTTCCTC
β-actin	AGCCATGTACGTCCATCC	CTCCAGCTGTGGTGGTGAA
Nkx6.1	CGCCCGGGCTCTACTTTAG	GTCCAGAGAACGTGGGTCTG
Pdx1	CTCCGGACATCTCCCCATAC	ACGGGTCCTCTTGTTTTCCT
MafA	ATCATCACTCTGCCCACCAT	AGTCGGATGACCTCCTCCTT

Rat primers

Gene	Froward primer	Reverse primer
ChREBPα	TGCATCGATCACAGGTCATT	AGGCTCAAGCATTCGAAGAG
ChREBPβ	TCTGCAGATCGCGCGGAG	CTTGTCCCGGCATAGCAA
Pklr	GTGGAGCACGGTGGTATCTT	CTTCACGCCTTCATGGTTCT
Txnip	CTGATGGAGGCACAGTGAGA	CTCGGGTGGAGTGCTTAGAG
β-actin	AACACCCCAGCCATGTACGTAG	GAACCGCTCATTGCCGATAGT

Genotyping primers

Mouse	Froward primer	Reverse primer
Flx ChREBPβ	GCTAGGGAGATGTGGCGTTT	GGTCCCAAACTGTAGACCGG
LSL ChREBPβ	AGGAAGACGAGGCTGGAGAT	TGACATCGATTACAAGGATGACGA
R26	GCAGAAGGAGCGGGAGAAAT	TTCCCCTCGTGATCTGCAAC
MIP Cre ERT	CGCGGTCTGGCAGTAAAAACTATC	CCCACCGTCAGTACGTGAGATATC
RIP Cre	TAAGGCTAAGTAGAGGTGT	TCCATGGTGATACAAGGGAC

Supplementary Table 2.

Name	Mono/ Polyclonal	Host species	Dilution Used	Supplier	Catalog #	Antibody Registry #
Insulin	Poly	Guinea Pig	1:000	Dako	A0564	AB_10013624
Glucagon	Mono	Mouse	1:500	Abcam	ab10988	AB_297642
ChREBP C term	Poly	Rabbit	1:250	Novus	NB400-135	AB_10002435
ChREBP N term		Rabbit	1:250	Genscript		
Flag	Poly	Rabbit	1:500	Cell Signaling	2368S	AB_2217020
GFP	Poly	Chicken	1:100	Aves-Labs	GFP-1020	AB_10000240
Cherry	Poly	Rabbit	1:1000	Rockland	600401379S	AB_11182807
Ki67	Mono	Rabbit	1:250	ThermoScientific	MA5-14520	AB_10979488

Supplementary Table 3 Human Islets used in this study:

T2D study (Figure 3b)

Unique identifiers	AACA334, AAKD226, ABAC372, ABHQ115, ABLM090, BALA1, H1714, H1732, HP14409, T2D4 / ABIC495	Aggregate Data
Origin, Islet isolation centers	IIDP, Prodo Labs, U Penn, Vanderbilt U., U Wis, U Edmonton,	
Donor age (years)		Range, 20-58; Average, 47.8 +/- 8.0 (N=8, SEM)
Donor sex (M/F)		4 F, 4 M, 2 Unknown
Donor BMI (kg/m2)		Range, 22.6-35.6: Average, 31.1 +/- 1.7 (N=7, SEM)
Donor history of diabetes? Yes/No		7 T2D, 4 No
Diabetes duration (years)		3-20 years

Txp localization (Figure 4f,g)

Unique identifiers	HP-16206, HP-2246, ADHR238A, HP-16250, HP-17055	Aggregate Data
Origin, Islet isolation centers	IIDP, Prodo Labs, U Miami, Southern California Islet Resource Center	
Donor age (years)		Range, 30-59; Average, 41.8 +/- 5.3; N=5, SEM
Donor sex (M/F)		1F, 4 M
Donor BMI (kg/m2)		Range, 25.1-34.1; Average, 30.5 +/- 1.7 (N=5, SEM)
Donor HbA1c or other measure of blood glucose control		Range 5.3-6.3, Average, 5.6 +/- 0.2 (N=5, SEM)
Estimated purity (%)		Range, 85-90, Average, 89.0 +/- 1.0 (N=5, SEM)
Estimated viability (%)		Range, 90-96, Average, 94.2 +/- 1.1 (N=5, SEM)
Total culture time (h)d		Range 43.5-192, Average, 99.9 +/- 25.0 (N=5, SEM)

Rescue (Figure 8d)

Unique identifiers	SAMN15770453, SAMN16427178, SAMN16515959	Aggregate Data
Origin, Islet isolation centers	IIDP, U Wis, Southern California Islet Resource Center	
Donor age (years)		Range, 48-51; Average, 47 +/- 2.6 (N=3, SEM)
Donor sex (M/F)		3F
Donor BMI (kg/m2)		Range, 25.2-31.2; Average, 29.1 +/- 2.0 (N=3, SEM)
Donor HbA1c or other measure of blood glucose control		Range, 5.5-5.8; Average, 5.6 +/- 0.1 (N=3, SEM)
Estimated purity (%)		Range, 80-90; Average, 86.7 +/- 3.3 (N=3, SEM)
Estimated viability (%)		Range, 96-99; Average, 97.0 +/- 1.0 (N=3, SEM)
Total culture time (h)d		Range 24-36; Average, 28.0 +/- 4.0 (N=3, SEM)

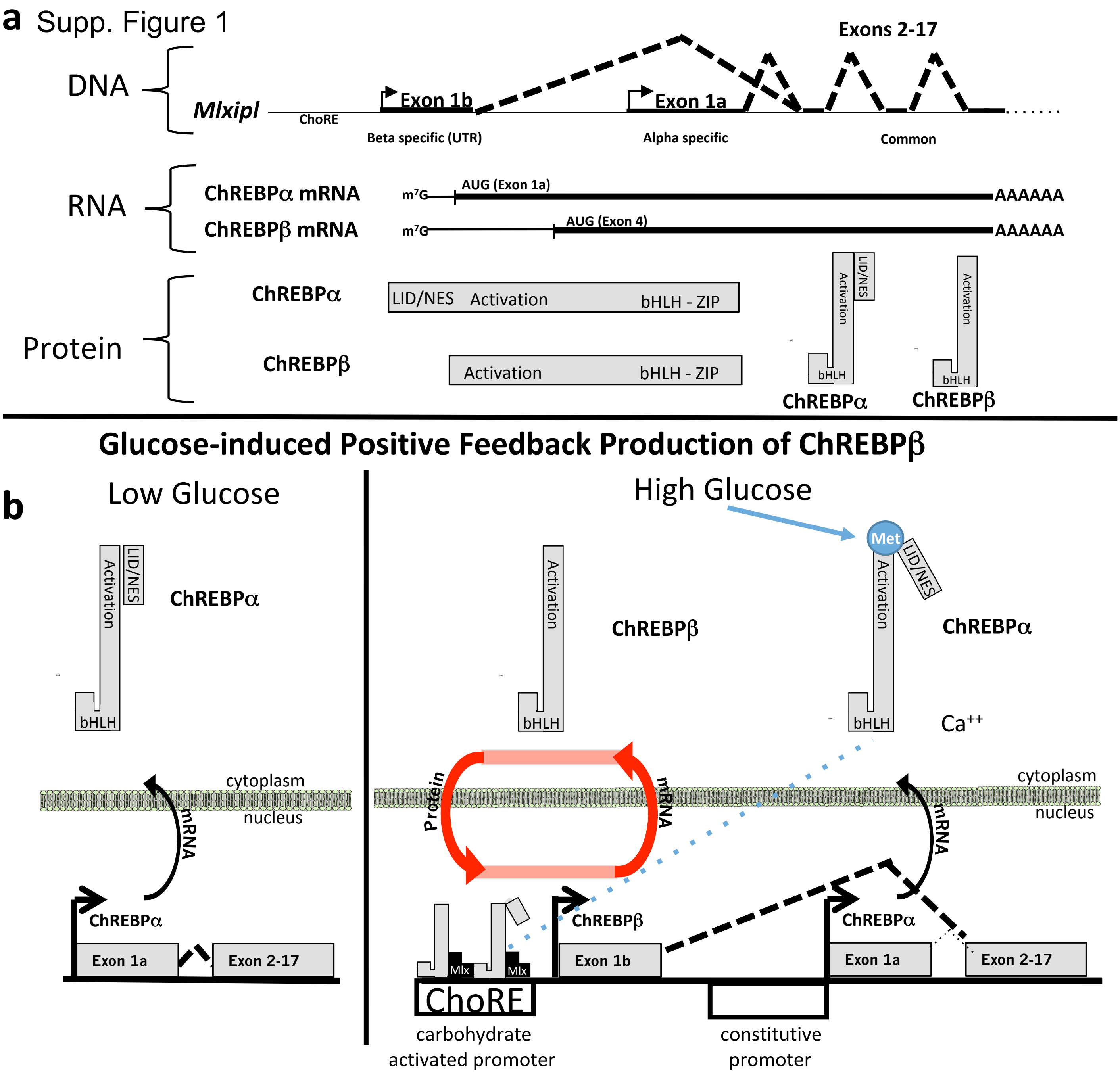

Supp. Figure 1. Production of ChREBPα and ChREBPβ. Two promoters of *Mlxipl* drive expression of the 2 major ChREBP isoforms, ChREBPα and ChREBPβ. The arrows denote transcription start sites. Alternative splicing results in mRNA species with different translation start sites and thus give rise to full length ChREBPα and a truncated ChREBPβ, without a low glucose inhibitory (LID) domain or nuclear export sequence (NES). The basic helix-loop-helix zipper (bHLH-Zip) domain allows for DNA binding and obligate heterodimerization with *Mlx*, respectively. The LID domain folds over and sterically inhibits the activation domain. ChREBPβ, without the LID or NES domains is much more transcriptionally active than ChREBPα. The alternative promoter for ChREBP contains a strong carbohydrate response element (ChoRE), setting the stage for a positive feedback loop. **b.** ChREBPα is constitutively produced and sequestered mostly in the cytoplasm under conditions of low glucose (*left panel*). With high glucose, a metabolite (Met) of glucose, whose identity is still controversial, binds to the glucose-sensing domain and a conformational change occurs, exposing the activation domain. Concurrently, in a Ca^{++}-dependent manner in β-cells, ChREBPα enters the nucleus and binds to the ChoRE. Alternative splicing leads to production of ChREBPβ, which immediately begins producing more of itself in a positive feedback loop (red, *right panel*).

Tools used to distinguish ChREBPα from ChREBPβ

1. RT-PCR primers specific to ChREBPα or ChREBPβ mRNA

2. Antibodies specific to ChREBPα (N-terminal) or both ChREBPα and ChREBPβ (C-terminal)

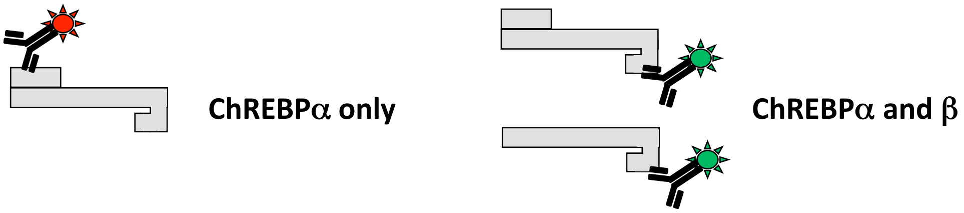

3. Anti-Flag antibodies specific to Flag-tagged ChREBPα or ChREBPβ

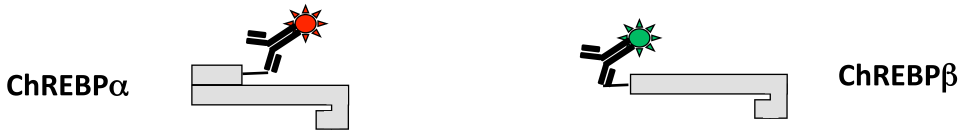

Supp. Figure 2. Tools used to distinguish ChREBPα from ChREBPβ. This figure lists tools we used in this study to distinguish between ChREBPα and ChREBPβ. **1.** RT-PCR was used with primers specific to either isoform. **2.** Antibodies against the N-terminal low glucose inhibitory domain (LID) can only recognize ChREBPα; antibodies against the C-terminus recognize both isoforms. **3.** Antibodies against the flag epitope tag can recognize flag-tagged ChREBPα or ChREBPβ.

Supp. Figure 3

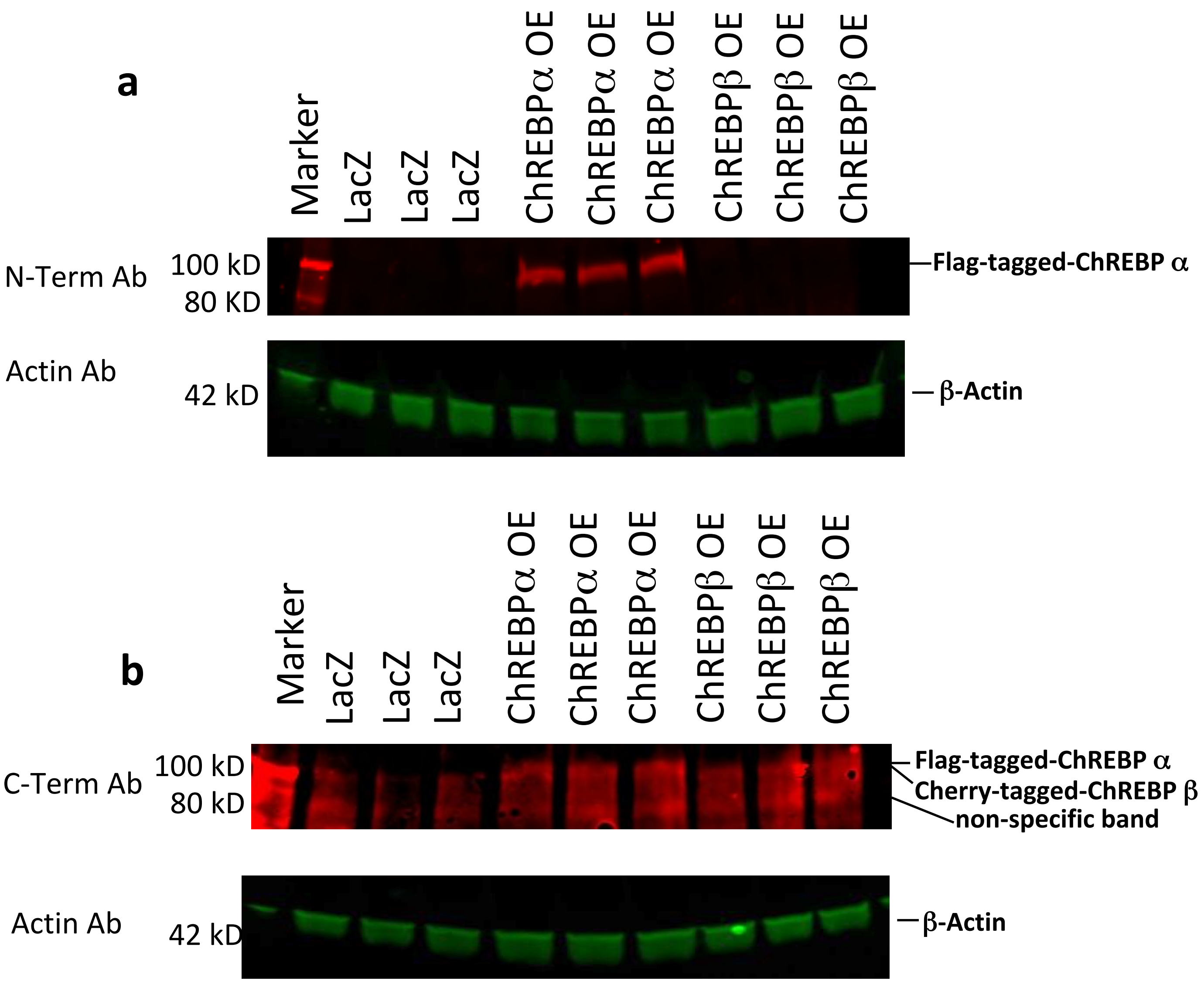

Supp. Figure 3. Validation of antibodies detecting ChREBP isoforms. INS-1 cells were transduced with the adenoviruses expressing either β-galactosidase (LacZ) as a control, or ChREBPα or ChREBPβ. Protein extracts were prepared, and Western blots performed using an N-terminal antibody to detect ChREBPα **(A)** and a C-terminal antibody to detect both ChREBPα and ChREBPβ **(B),** and a-Actin as a loading control. Note that Cherry-tagged ChREBPβ migrates at nearly the same rate as Flag-tagged ChREBPα. Shown are the extracts from 3 separate experiments.

Supp. Figure 4

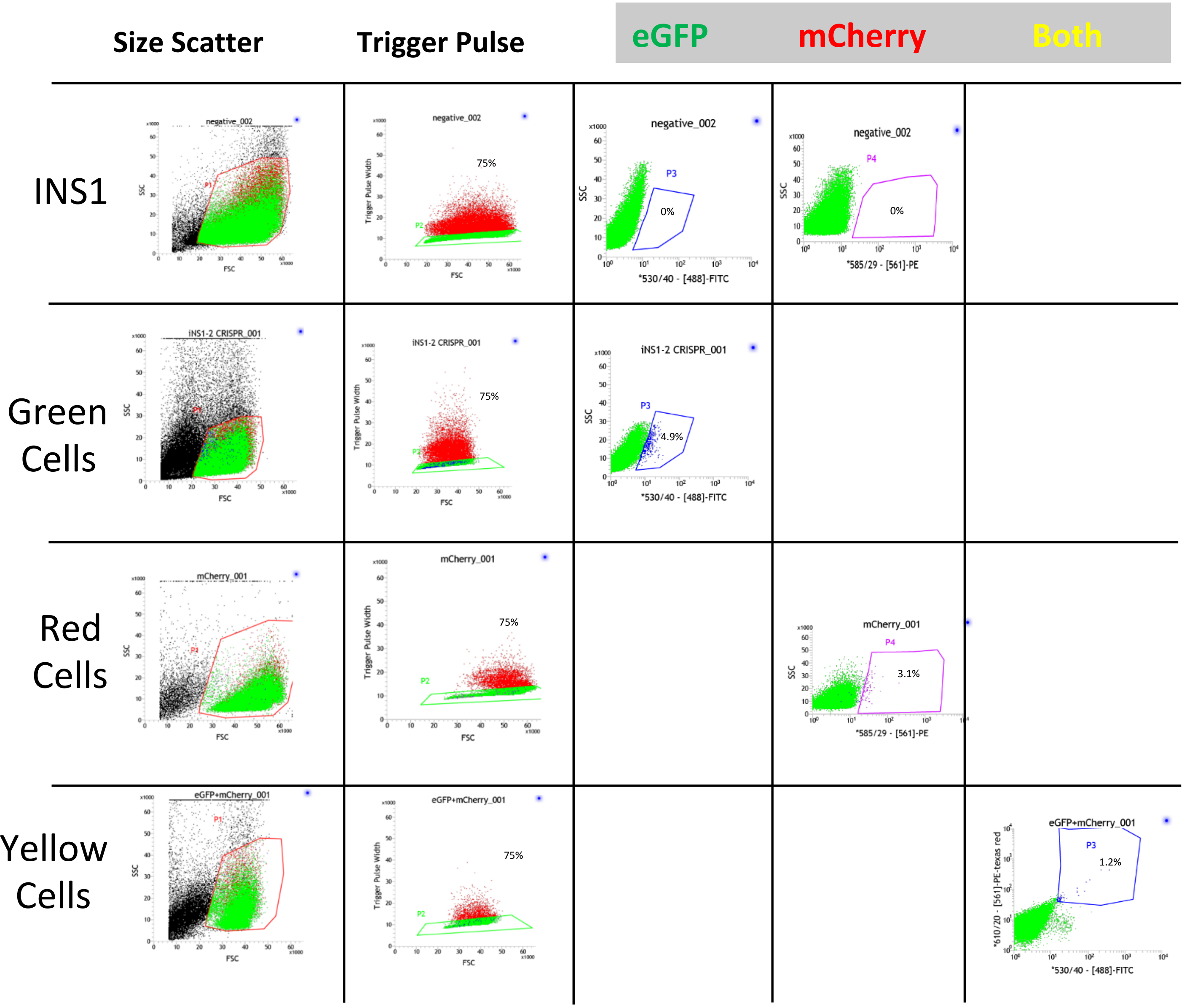

Supp. Figure 4. Gating and fluorescent activated cell sorting of ChREBP genomically edited with eGFP and mCherry labels in INS-1 cells. INS-1 cells were genome-edited using CRISPR/Cas9 as described in the Methods to add GFP or Cherry tags or both to the endogenous Mlxipl (ChREBP) gene (and see Suppl. Fig 1). Cells were sorted on a FACSAria II in the Mount Sinai Flow Cytometery Core. Shown is a grid with the gating used to sort the edited INS-1 cells to derive Green, Red and Red/Green INS-1 cells. The data shown represents the first of several rounds of sorting.

Supp. Figure 5

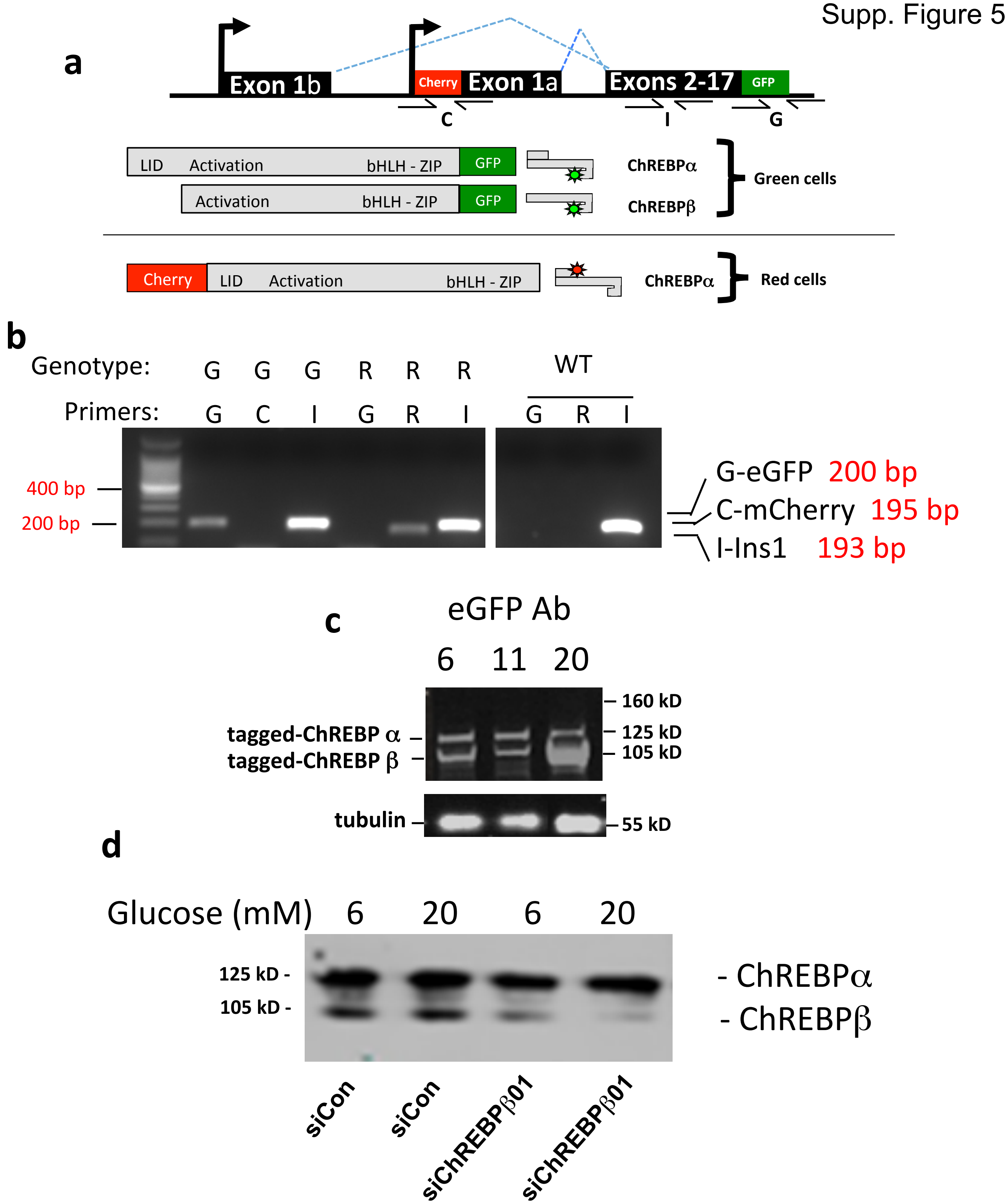

Supp. Figure 5. Validation of Green and Red ChREBP-edited INS-1 cells. a. Schematic of genomic editing of ChREBP including location of genotyping primers. **b.** Agarose gel of genotyping red and green cells using the indicated primers from **(a). c.** Western blot of extracts from Green (eGFP antibody) cells treated with the indicated concentration of glucose for 16 h. β-Actin was used as loading controls. **d.** Western blot from extracts of Green cells treated with a control siRNA or and siRNA against ChREBPβ and cultured for 2 days in the indicated glucose concentration.

Supp. Figure 6

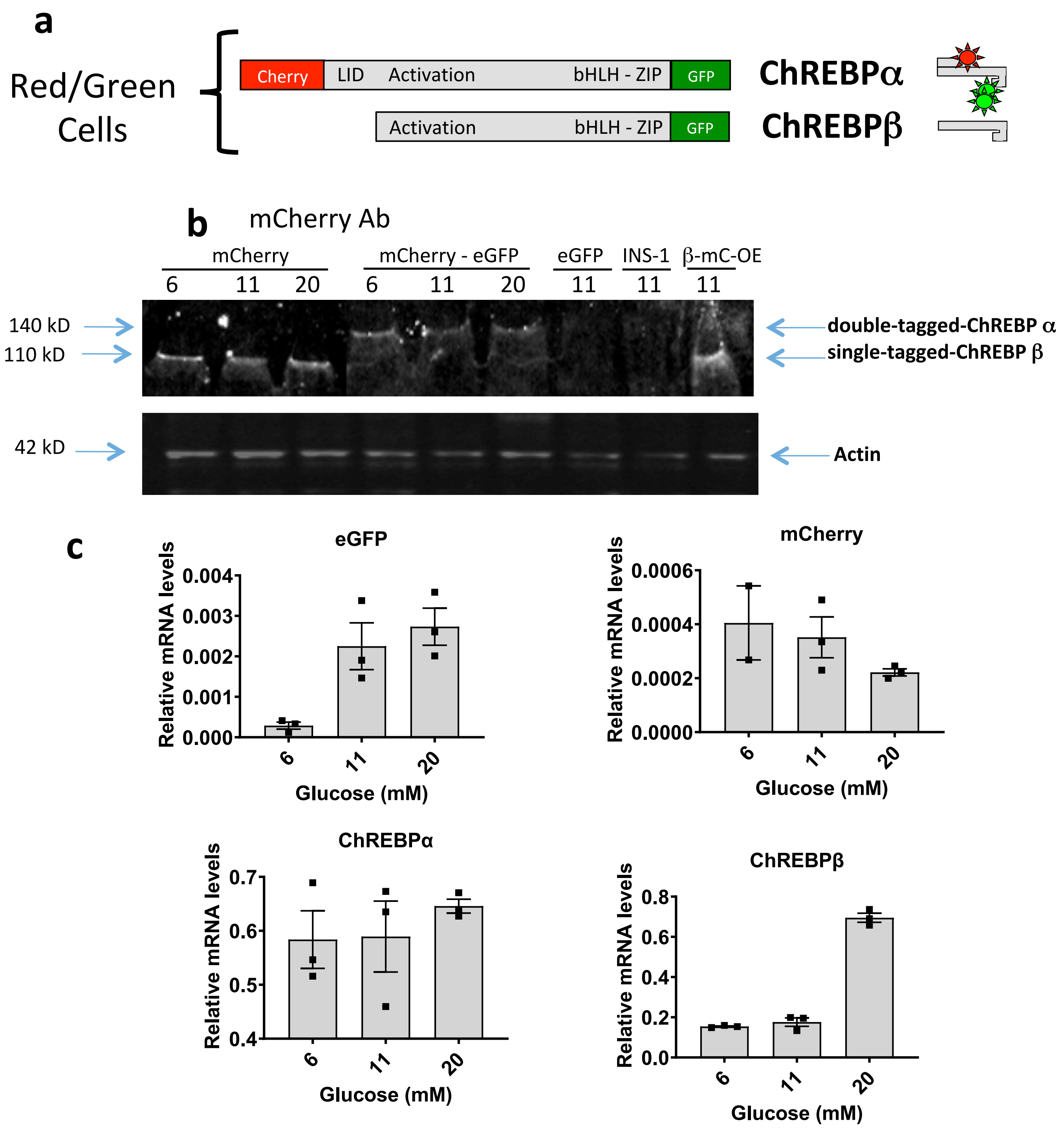

Supp. Figure 6. Validation of Red and Red/Green ChREBP-edited INS-1 cells. a. Schematic of genomic editing of Red/Green cells. **b.** Western blot of protein extracts of Red (mCherry), Green (eGFP), Red/Green (mCherry-eGFP), or parental INS1 cells cultured for 2 days in 6, 11, or 20 mM glucose. **c.** RNA was isolated from Red/Green cells cultured in the indicated concentrations of glucose and subjected to RT-PCR using primers for the indicated genes. Data are the means +/- SEM, n=3.

Supp. Figure 7

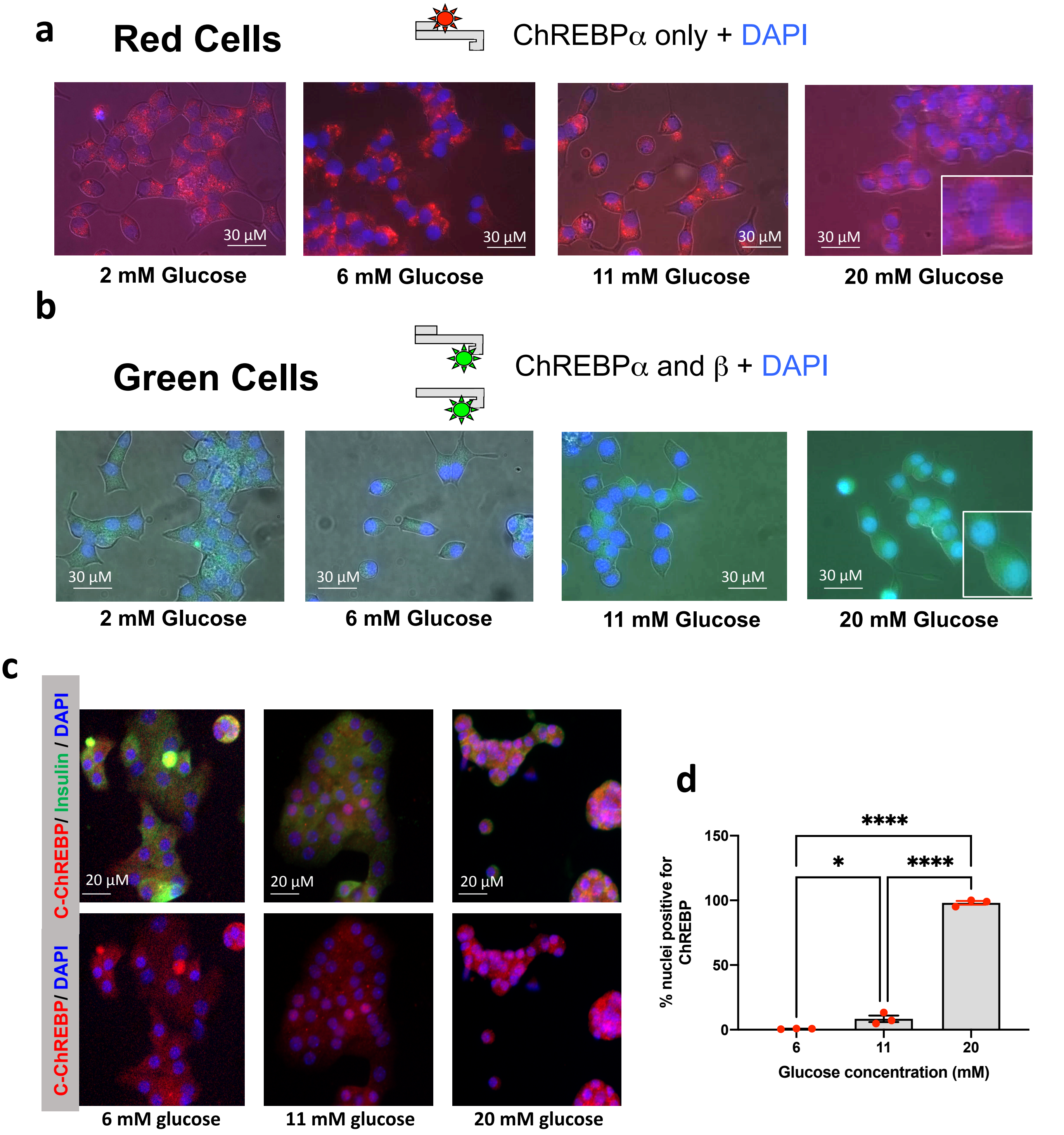

Supp. Figure 7. ChREBPβ becomes more nuclear with increasing concentrations of glucose. Red or Green INS-1 cells were incubated in the indicated concentrations of glucose for 72 h. **a,b.** Live cell microscopy was performed, which includes phase contrast to identify the edges of the cells. All micrographs represent at least 3 independent experiments. **c.** Islets from male C57Bl/6 mice were isolated, dispersed and cultured in the indicated glucose concentrations for 48 h. Cells were fixed and immunostained with the C-terminal antibody for ChREBP (recognizes both ChREBPα and ChREBPβ, stained red) and Insulin (stained green) **d.** The percent nuclear ChREBP fluorescence from **(C)** was determined. Data are the means +/- SEM, N=3, *, $p < 0.05$, ****. $P<0.0001$ using two-way ANOVA.

Supp. Figure 8

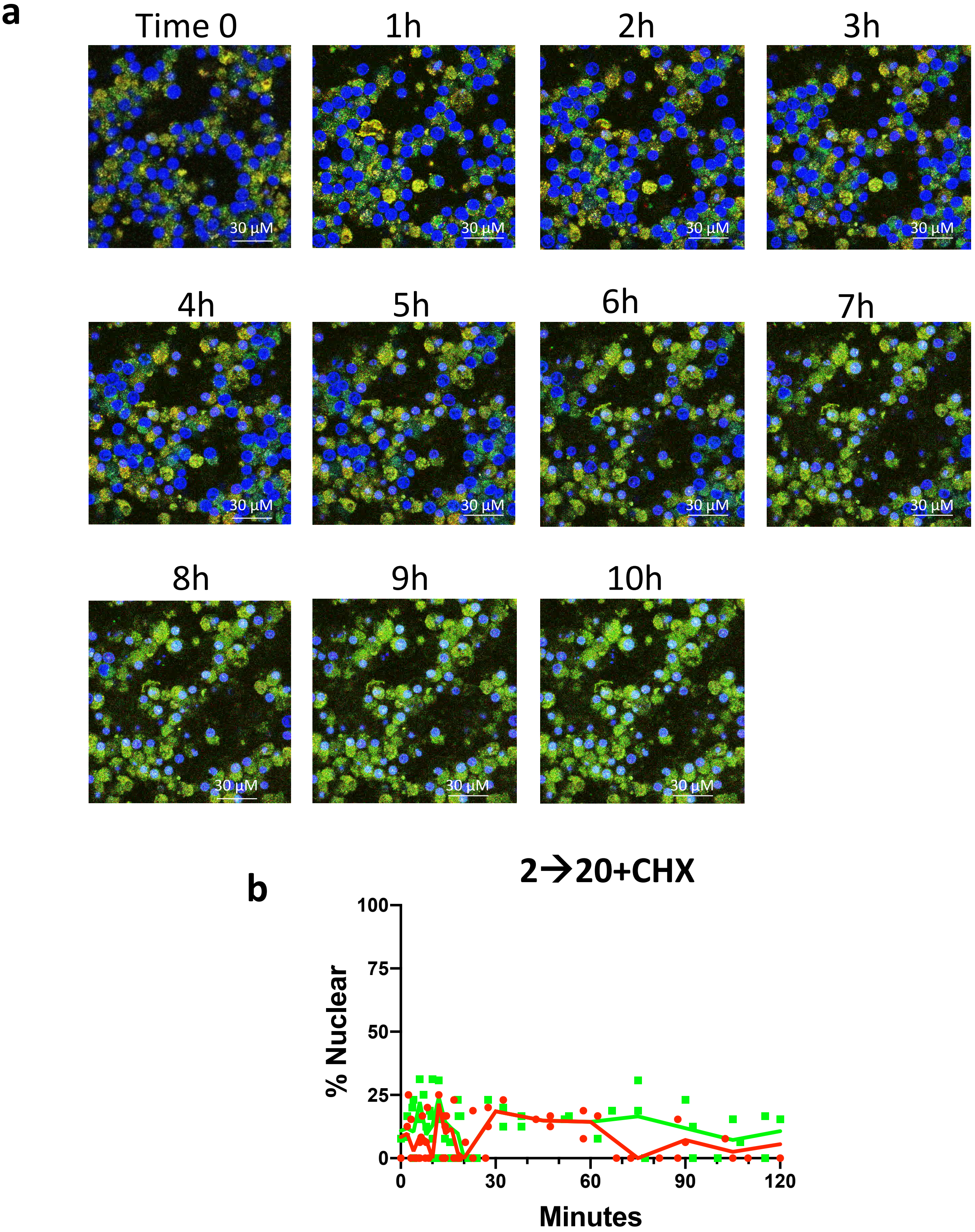

Supp. Figure 8. Time course of Red/Green cells after changing media from 2 mM to 20 mM glucose. **a.** A field of Yellow (Red/Green) fluorescence from 0 to10 h after changing culture medium from 2 mM to 20 mM glucose. **b.** Percent nuclear red or green fluorescence after changing culture medium from 2 to 20 mM glucose in the presence of 10µg/ml cycloheximide (CHX). Representative of 3 independent experiments.

Supp. Figure 9

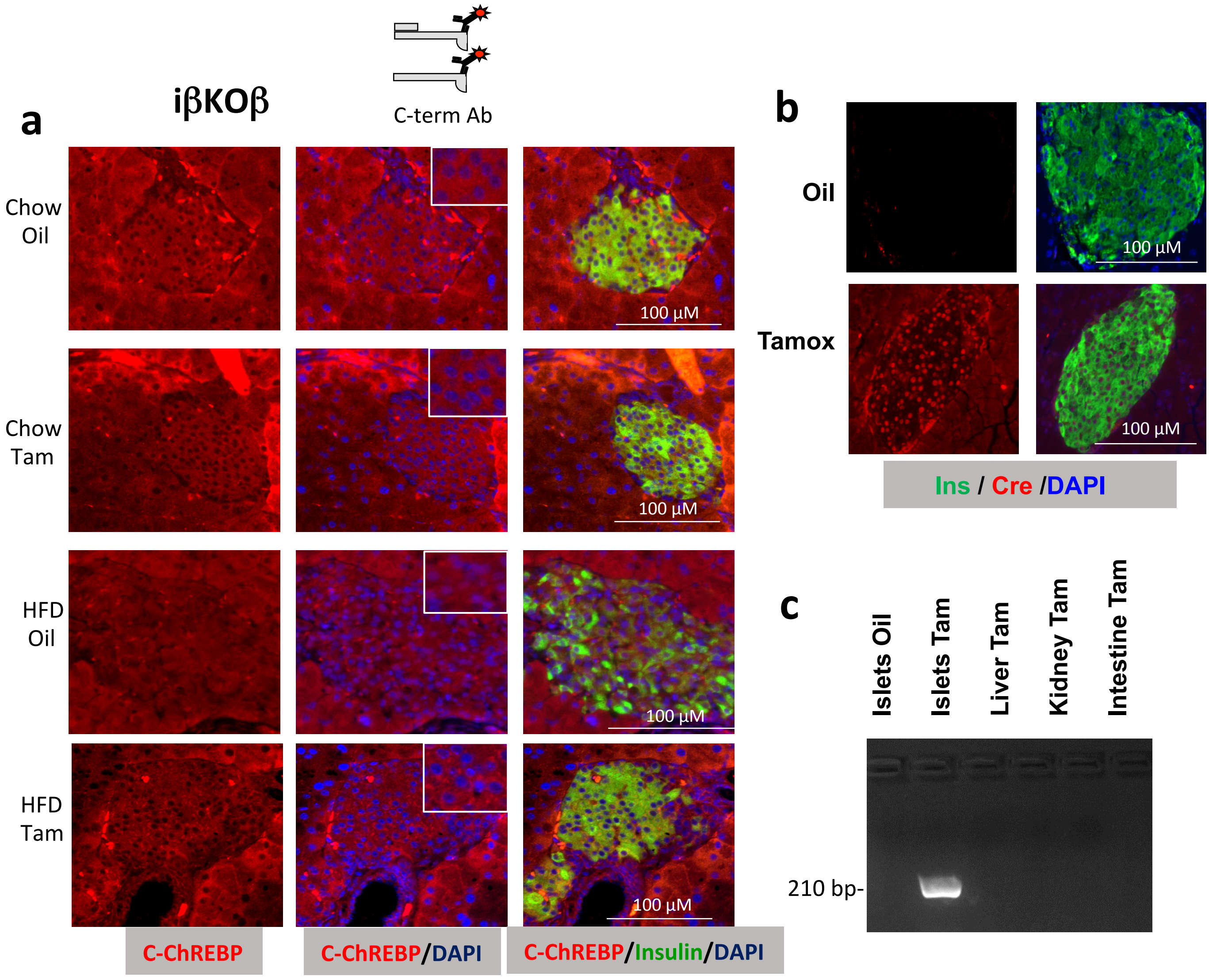

Supp. Figure 9. Validation of iβKOβ mice. a. iβKOβ mice were treated with vehicle corn oil or tamoxifen, allowed to recover for 2 days and then placed on a chow or high-fat diet (HFD) for one week. Pancreata were harvested and tissue slices were immunostained with antibodies against insulin or C-terminal ChREBP. **b.** iβKOβ mice were treated with vehicle corn oil or tamoxifen. After 7 days past the first injection, pancreata were harvested and immunostained for Cre recombinase and insulin. **c.** iβKOβ mice were treated with vehicle corn oil or tamoxifen, allowed to recover for 2 days and DNA from the indicated tissues was isolated and used as template for a PCR reaction that provided an amplicon only after recombination resulting in the deletion of ChREBP intron 1b. These results were repeated at least 3 independent times.

Supp. Figure 10

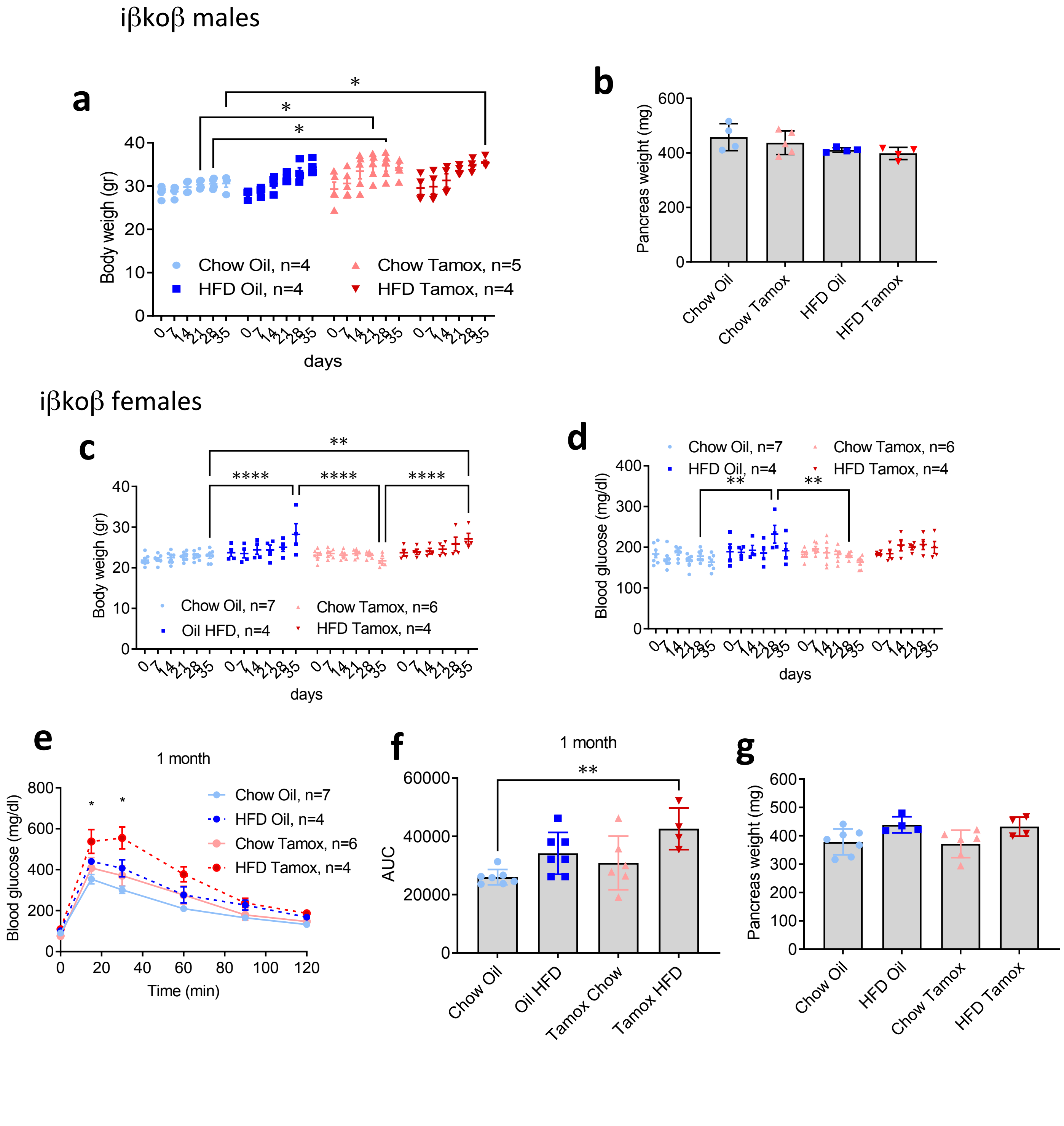

Supp. Figure 10. Female iβKOβ mice are largely protected from the loss of ChREBPβ. a. Male iβKOβ body weight. **b.** Male iβKOβ weight of pancreata at time of euthanasia. **c.** Female iβKOβ body weight. **d.** Female iβKOβ blood glucose levels. **e,f.** Glucose tolerance test and area under the curve in female oil- or tamoxifen-treated iβKOβ mice **g.** Female iβKOβ weight of pancreata. Data are the means +/- SEM, N=4-7, *, $p < 0.05$, **, $p < 0.01$ using two-way ANOVA.

Supp. Figure 11

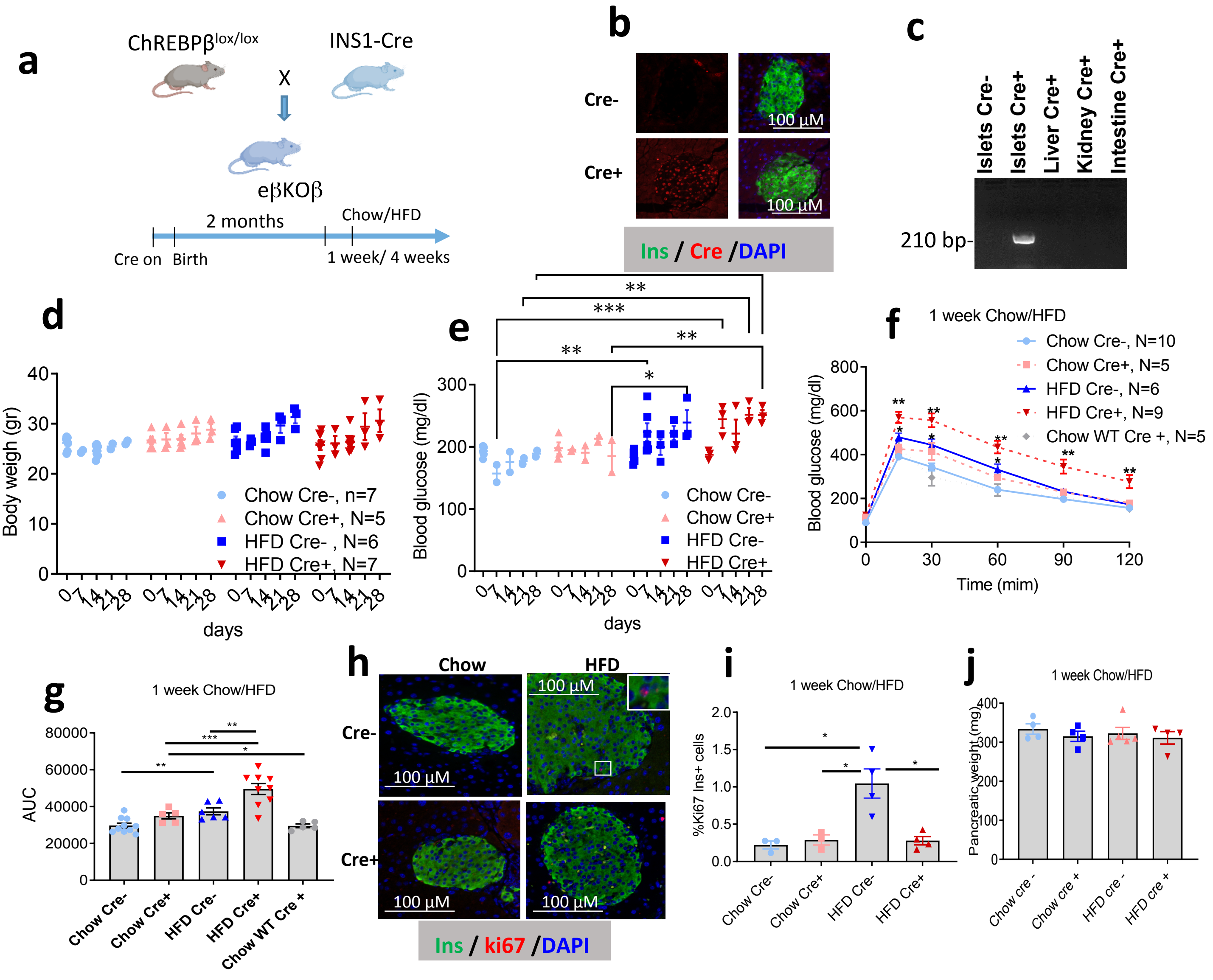

Supp. Figure 11. ChREBPβ is necessary for adaptive β-cell proliferation. a. Schematic showing β-cell specific embryonic knockout of ChREBPβ in mice (eβKOβ, created with BioRender.com). **b.** Pancreata from 8-week-old Cre positive or negative eβKOβ mice were harvested and immunostained for Cre recombinase and insulin. **c.** DNA from the indicated tissues was isolated from Cre positive or negative eβKOβ mice and used as template for a PCR reaction that provided an amplicon only after deletion of ChREBP intron 1b. **d.** Body weight of Cre positive or negative eβKOβ mice after the indicated time and diets. **e.** Blood glucose levels after the indicated treatments and times. **f,g.** Glucose tolerance test and area under the curve measurements after 1 week on a chow or high-fat diet (HFD). **h,i.** Percent Ki67-postive and insulin-positive cells in pancreata from eβKOβ mice after 1 week of HFD. **j.** Pancreatic weight after 1 week on a HFD. Data are the means +/- SEM, N=4-7, *, $p < 0.05$, **, ***, $p<0.005$ using two-way ANOVA.

Supp. Figure 12

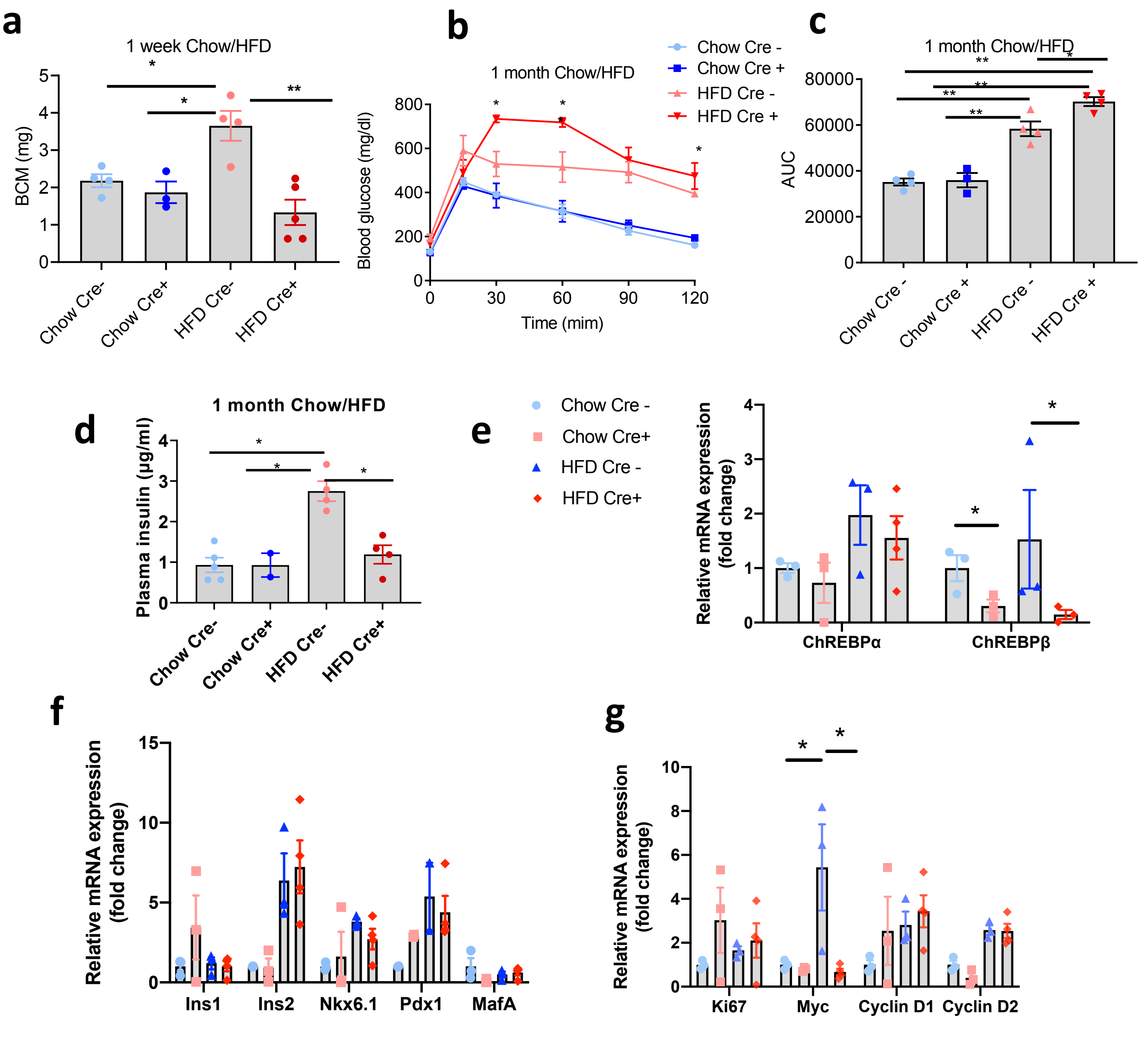

Supp. Figure 12. ChREBPβ is necessary for adaptive expansion of β-cell mass, but dispensable for β-cell development a. β-cell mass after one week on a chow or HFD. **b,c.** Glucose tolerance test and area under the curve measurements after 1 month on a chow or high-fat diet (HFD). **d.** Plasma insulin after 1 month on a HFD. **e-g.** Relative abundance of the indicated genes normalized to β-actin as determined by RT-PCR. Data are the means +/- SEM, N=4-7, *, $p < 0.05$, **, ***, $p<0.005$ using two-way ANOVA.

Supp. Figure 13

Female eβKOβ mice

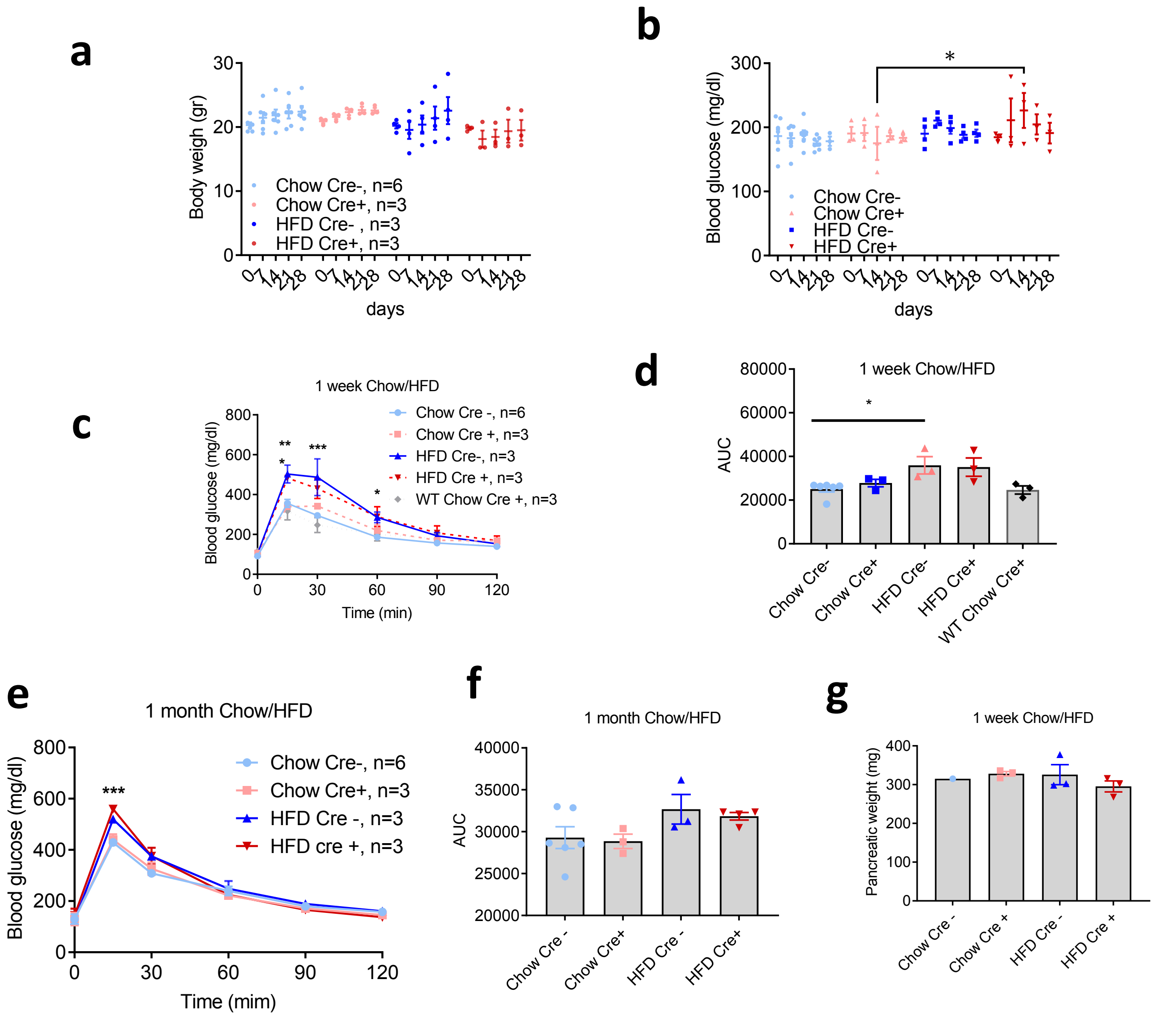

Supp. Figure 13. Female eβKOβ mice are largely protected from the loss of ChREBPβ. a. Female eβKOβ body weight after the indicated diets and time. **b**. Female eβKOβ blood glucose levels. **c,d.** Glucose tolerance test and area under the curve in female eβKOβ mice at 8 weeks of age after 1 week of chow or HFD. **e,f.** Glucose tolerance test and area under the curve in female eβKOβ mice after 1 month of Chow or HFD **g.** Weight of pancreata from female eβKOβ at 1 month. Data are the means +/- SEM, N=3-5, *, $p < 0.05$, **, $p < 0.01$ using two-way ANOVA.

Supp. Figure 14

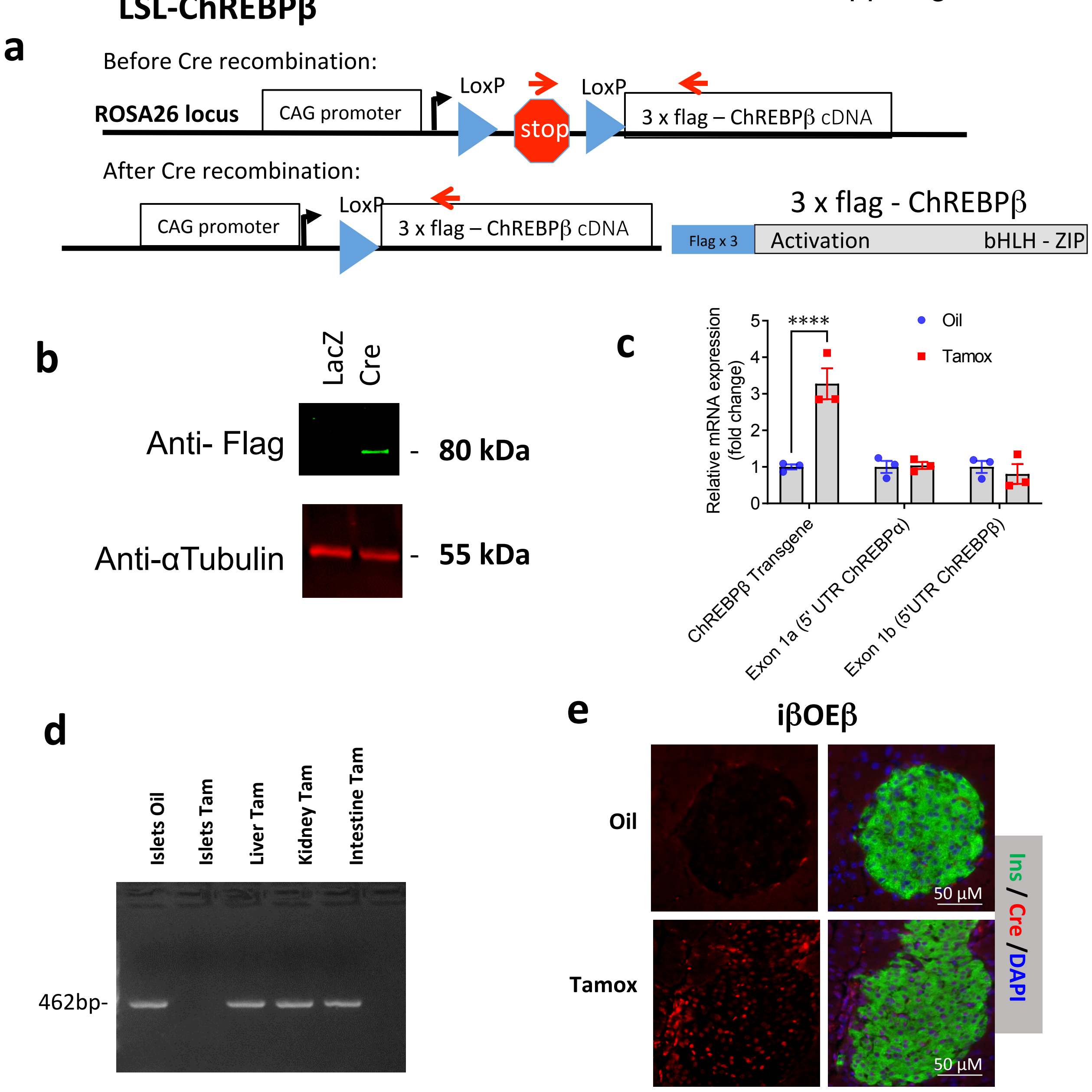

Supp. Figure 14. Validation of LSL-ChREBPβ and iβOEβ mice. a. Schematic showing Cre-mediated recombination of LoxP – Stop codon – LoxP (LSL)-ChREBPβ mice resulting in expression of flag-tagged ChREBPβ. **b**. Western blot showing expression of flag-tagged ChREBPβ from isolated islets from LSL-ChREBPβ mice after transduction with adenovirus expressing control β-galactosidase (LacZ) or Cre. **c.** qRT-PCR of mRNA expression of the flag-tagged-ChREBPβ transgene or endogenous ChREBPα or ChREBPα normalized to β-actin isolated islets from iβOEβ mice treated as indicated. Data are the means +/- SEM, n=3, ****, P<0.0001 using two-way ANOVA. **d**. iβOEβ mice were treated with vehicle corn oil or tamoxifen, allowed to recover for one week and DNA from the indicated tissues was isolated and used as template for a PCR reaction that failed to provide an amplicon after recombination resulting in the deletion of ChREBP intron 1b. **e.** iβOEβ mice were treated with vehicle corn oil or tamoxifen. After one week past the first injection, pancreata were harvested and immunostained for Cre recombinase and insulin. The results are representative of 3 independent experiments.

Supp. Figure 15

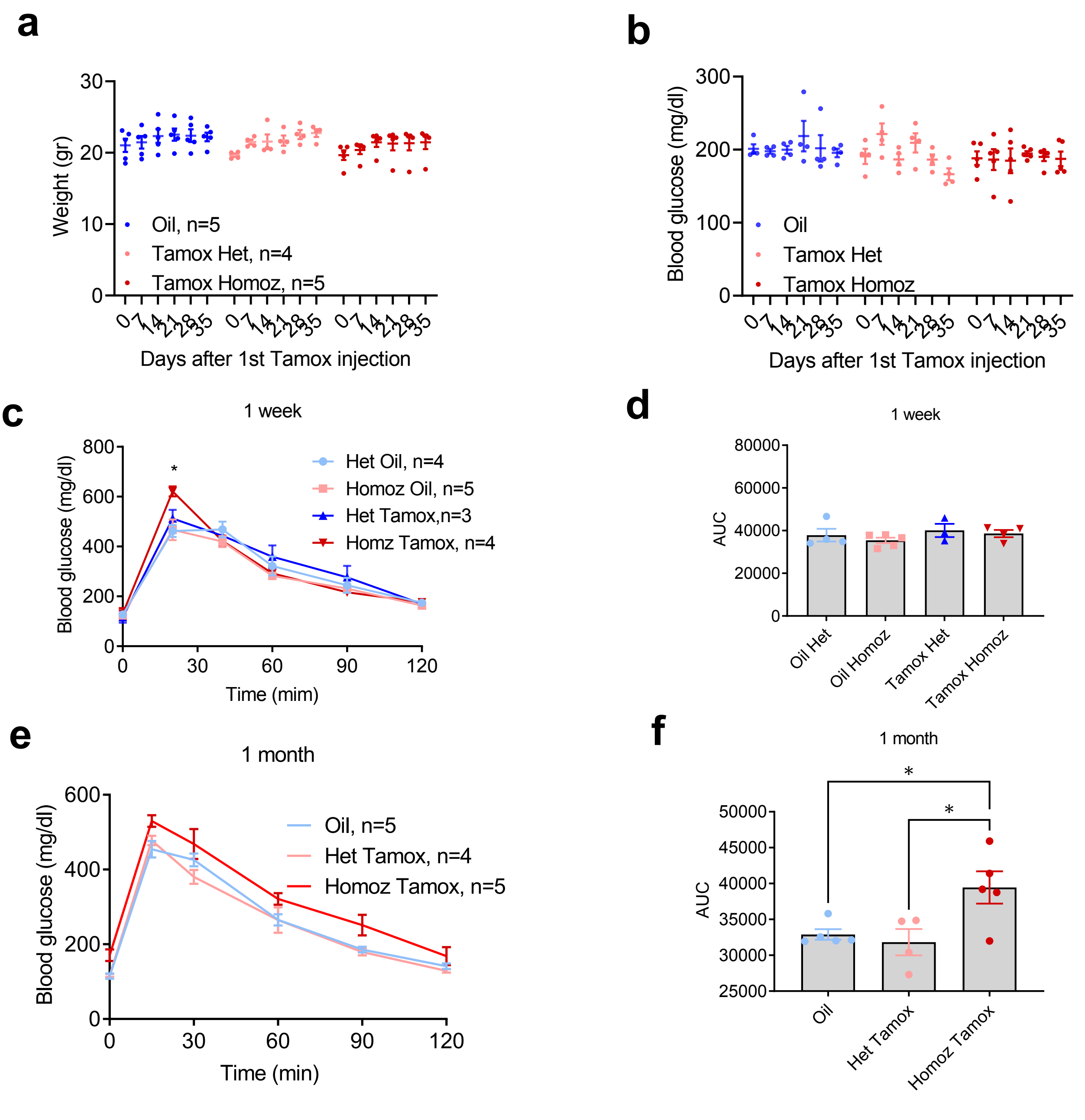

Supp. Figure 15. Female iβOEβ mice are largely protected from overexpression of ChREBPβ. a. Body weight of control (oil-treated) or heterozygous or homozygous female iβOEβ after tamoxifen treatment flowed by the indicated times. **b.** Heterozygous or homozygous female iβOEβ blood glucose levels after oil or tamoxifen treatment. **c,d.** Glucose tolerance test and area under the curve in female heterozygous or homozygous iβOEβ mice one week after the last injection with oil or tamoxifen. **e,f.** Glucose tolerance test and area under the curve (AUC) in female heterozygous or homozygous iβOEβ mice one month after the last injection with oil or tamoxifen. Data are the means +/- SEM, n=4-5, *, $p < 0.05$ using two-way ANOVA.

Supp. Figure 16

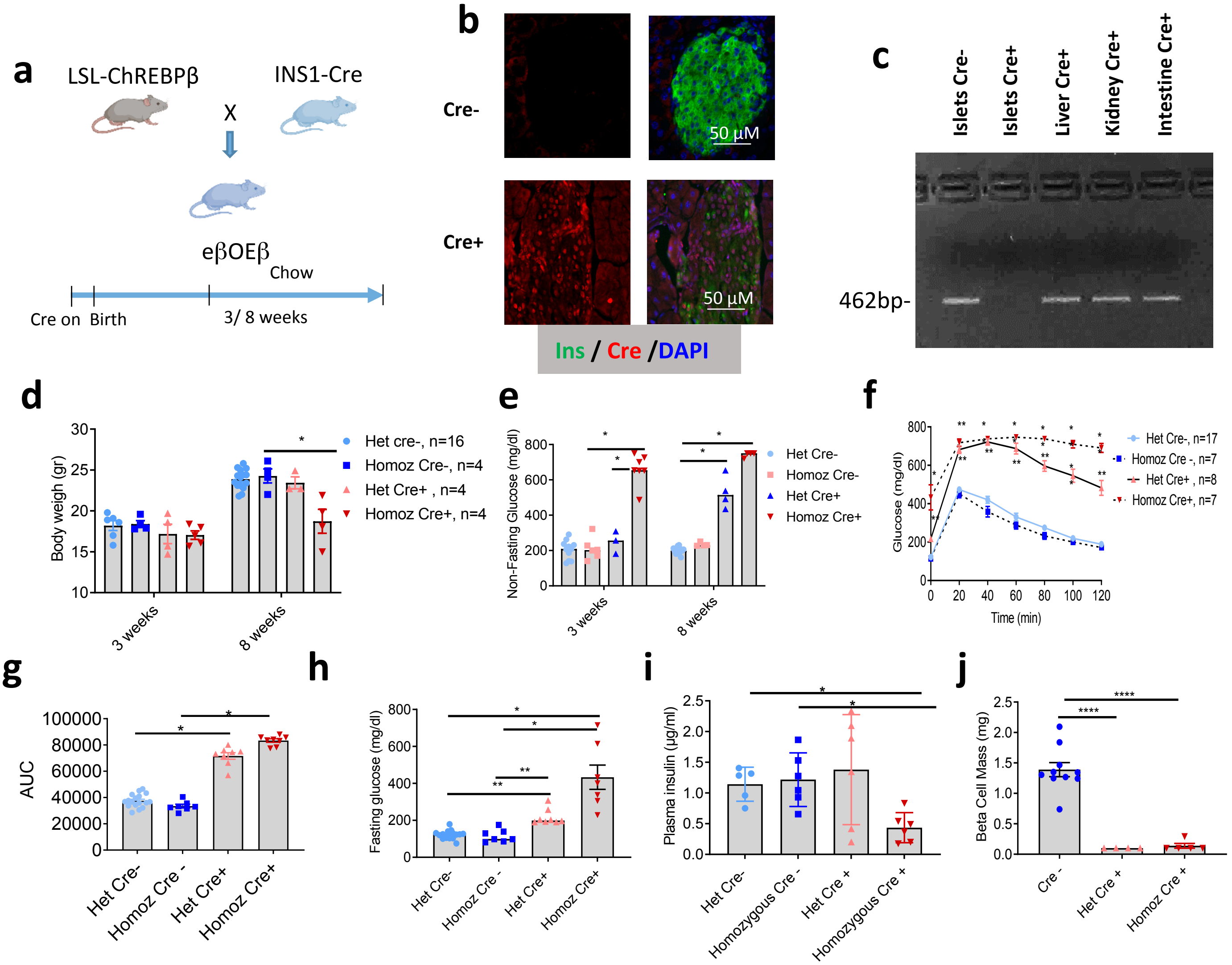

Supp. Figure 16. Embryonic overexpression of ChREBPβ leads to β-cell death, glucose intolerance, and diabetes in male mice. a. Schematic showing β-cell specific embryonic overexpression of ChREBPβ in mice (eβOEβ, created with BioRender.com). **b.** Pancreata from 8-week-old Cre positive or negative eβOEβ mice were harvested and immunostained for Cre recombinase and insulin. **c.** Tissue from 8-week-old eβOEβ mice was collected and DNA from the indicated tissues was isolated and used as template for a PCR reaction that failed to provide an amplicon after recombination resulting in the deletion of ChREBP intron 1b. **d.** Body weight of Cre positive or negative eβKOβ mice after the indicated time and diets. **e.** Non-fasting blood glucose levels in male heterozygous or homozygous Cre positive or negative eβOEβ mice at 3 weeks and 8 weeks of age. **f,g.** Glucose tolerance test and area under the curve measurements of male heterozygous or homozygous Cre positive or negative eβOEβ mice at 8 weeks of age. **h-j.** Fasting blood glucose levels, plasma insulin, and β-cell mass measurements from male heterozygous or homozygous Cre positive or negative eβOEβ mice at 8 weeks of age. Data are the means +/- SEM, N≥3, *, $p < 0.01$. **, $p < 0.05$ using two-way ANOVA, All micrographs represent at least 3 different mice.

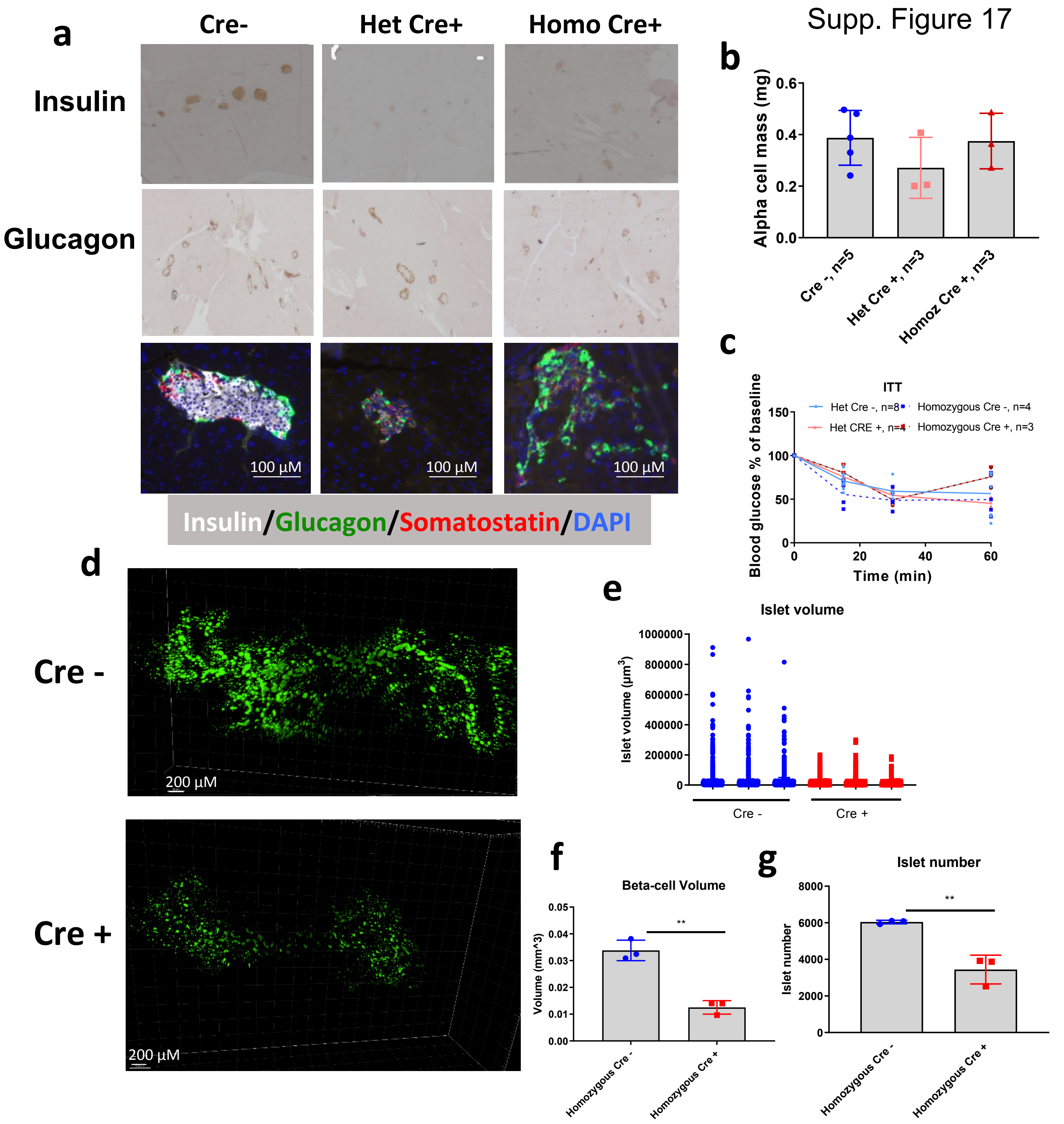

Supp. Figure 17. a. Immunochemical or immunofluorescent staining of pancreata using antibodies against insulin, glucagon or somatostatin from male heterozygous or homozygous Cre positive or negative eβOEβ mice at 8 weeks of age. **b.** Alpha cell mass from the same tissue preparations in K. **c.** Insulin tolerance test of male eβOEβ mice with the indicated genotypes. **d.** Pancreata were perfused and isolated from Cre positive or negative male eβOEβ mice and clarified using the iDISCO method, and immunolabeled with an antibody against insulin. Lightsheet images were acquired. **e-g** Imaris was used calculate islet volume, β-cell volume and islet number from N. Data are the means +/- SEM, N≥3, *, $p < 0.01$. **, $p < 0.05$ using two-way ANOVA, All micrographs represent at least 3 different mice.

Supp. Figure 18

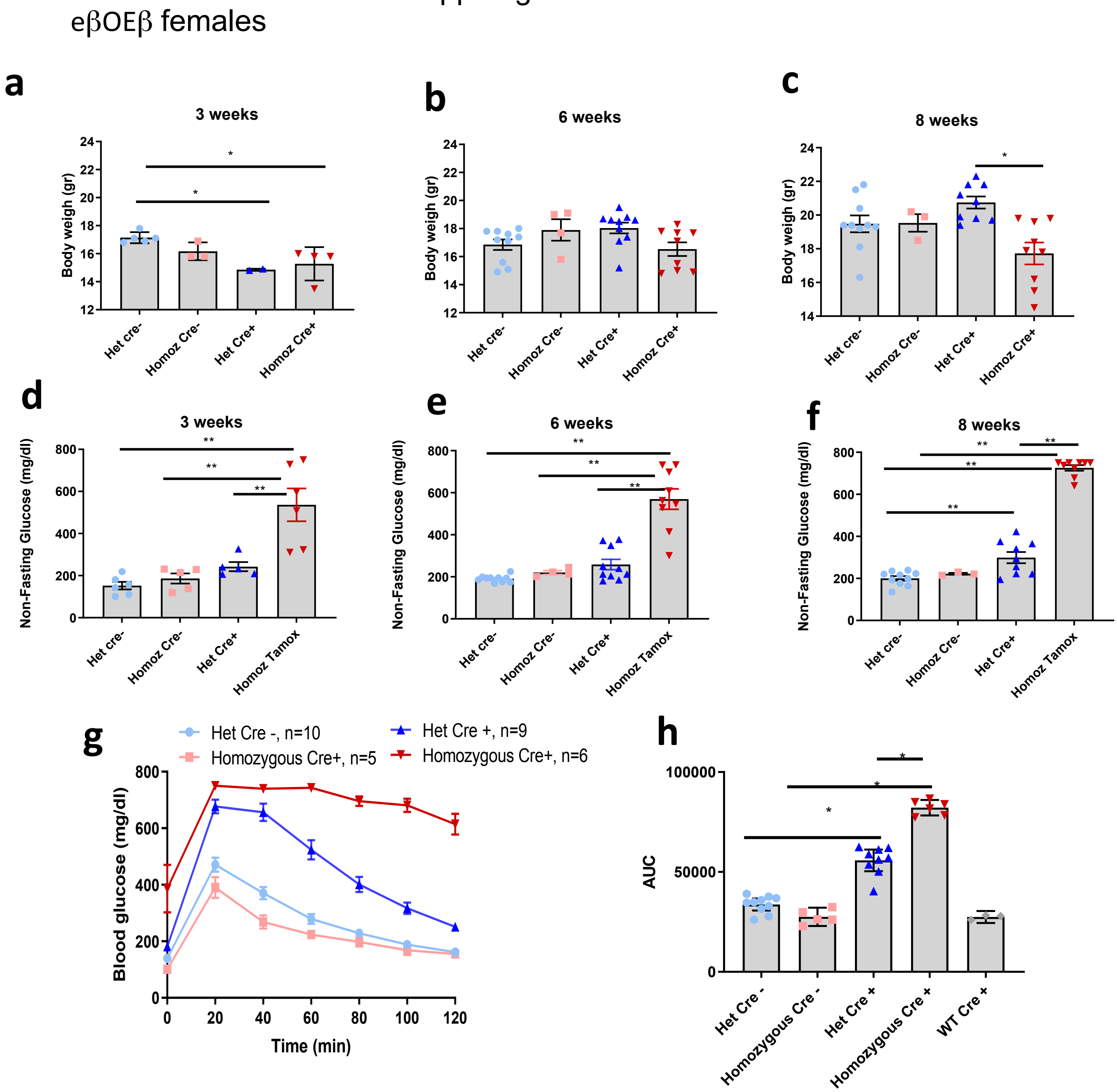

Supp. Figure 18. Embryonic overexpression of ChREBPβ leads to β-cell death, glucose intolerance, and diabetes in female mice. a-c. Body weight of Cre positive or Cre negative heterozygous or homozygous female eβOEβ mice at 3, 6 or 8 weeks of age. **d-f.** Non-fasting blood glucose levels in Cre positive or Cre negative heterozygous or homozygous female eβOEβ mice at 3, 6 or 8 weeks of age. **g, h.** Glucose tolerance test and area under the curve (AUC) for the indicated genotypes. Data are the means +/- SEM, N=5-10, *, $p < 0.05$. *, $p < 0.05$, **, $p < 0.01$ using two-way ANOVA,
