# Supplementary material for: Maladaptive positive feedback production of ChREBPβ underlies glucotoxic β-cell failure
Source: Nat Commun. 2022 Jul 30;13:4423. doi: 10.1038/s41467-022-32162-x (PMC9339008; doi:10.1038/s41467-022-32162-x)
Supplement: Supplementary file 2 — Reporting Summary [file 41467_2022_32162_MOESM2_ESM.pdf]

nature portfolio

Corresponding author(s): Donald K. Scott

Last updated by author(s): Jun 6, 2022

Reporting Summary

Nature Portfolio wishes to improve the reproducibility of the work that we publish. This form provides structure for consistency and transparency in reporting. For further information on Nature Portfolio policies, see our Editorial Policies and the Editorial Policy Checklist.

Statistics

For all statistical analyses, confirm that the following items are present in the figure legend, table legend, main text, or Methods section.

n/a	Confirmed	
☐	☒	The exact sample size (*n*) for each experimental group/condition, given as a discrete number and unit of measurement
☐	☒	A statement on whether measurements were taken from distinct samples or whether the same sample was measured repeatedly
☐	☒	The statistical test(s) used AND whether they are one- or two-sided<br>*Only common tests should be described solely by name; describe more complex techniques in the Methods section.*
☐	☒	A description of all covariates tested
☒	☐	A description of any assumptions or corrections, such as tests of normality and adjustment for multiple comparisons
☐	☒	A full description of the statistical parameters including central tendency (e.g. means) or other basic estimates (e.g. regression coefficient) AND variation (e.g. standard deviation) or associated estimates of uncertainty (e.g. confidence intervals)
☐	☒	For null hypothesis testing, the test statistic (e.g. *F*, *t*, *r*) with confidence intervals, effect sizes, degrees of freedom and *P* value noted<br>*Give P values as exact values whenever suitable.*
☒	☐	For Bayesian analysis, information on the choice of priors and Markov chain Monte Carlo settings
☒	☐	For hierarchical and complex designs, identification of the appropriate level for tests and full reporting of outcomes
☒	☐	Estimates of effect sizes (e.g. Cohen's *d*, Pearson's *r*), indicating how they were calculated

Our web collection on statistics for biologists contains articles on many of the points above.

Software and code

Policy information about availability of computer code

Data collection	Imaris version 9.1.0 was used to collect data from light sheet microscopy.
Data analysis	Graphpad Prism version 9.3.1 was used for statistical analysis and for making graphs. Excell for Mac version 16.54 was used to make some graphs. Viseago version 1.8.0 and the edgeR R package version 4.2 were used for analysis of RNAseq data.

For manuscripts utilizing custom algorithms or software that are central to the research but not yet described in published literature, software must be made available to editors and reviewers. We strongly encourage code deposition in a community repository (e.g. GitHub). See the Nature Portfolio guidelines for submitting code & software for further information.

Data

Policy information about availability of data

All manuscripts must include a data availability statement. This statement should provide the following information, where applicable:
- Accession codes, unique identifiers, or web links for publicly available datasets
- A description of any restrictions on data availability
- For clinical datasets or third party data, please ensure that the statement adheres to our policy

The datasets generated during and/or analysed during the current study are available in the GEO repository as accession number GSE197864 (https://www.ncbi.nlm.nih.gov/geo/query/acc.cgi?acc=GSE197864).

Human research participants

Policy information about studies involving human research participants and Sex and Gender in Research.

Reporting on sex and gender	*Use the terms sex (biological attribute) and gender (shaped by social and cultural circumstances) carefully in order to avoid confusing both terms. Indicate if findings apply to only one sex or gender; describe whether sex and gender were considered in study design whether sex and/or gender was determined based on self-reporting or assigned and methods used. Provide in the source data disaggregated sex and gender data where this information has been collected, and consent has been obtained for sharing of individual-level data; provide overall numbers in this Reporting Summary. Please state if this information has not been collected. Report sex- and gender-based analyses where performed, justify reasons for lack of sex- and gender-based analysis.*
Population characteristics	*Describe the covariate-relevant population characteristics of the human research participants (e.g. age, genotypic information, past and current diagnosis and treatment categories). If you filled out the behavioural & social sciences study design questions and have nothing to add here, write "See above."*
Recruitment	*Describe how participants were recruited. Outline any potential self-selection bias or other biases that may be present and how these are likely to impact results.*
Ethics oversight	Human beta cells were isolated from human cadaveric islets donors provided by the NIH/NIDDK-supported Integrated Islet Distribution Program (IIDP) (https://iidp.coh.org/overview.aspx), and from Prodo Labs (https://prodolabs.com/), the University of Miami, the University of Minnesota, the University of Wisconsin, the Southern California Islet Cell Resource enter, and the University of Edmonton, as summarized in Supplementary Table 3. Informed consent was obtained by the Organ Procurement Organization (OPO), and all donor information was de-identified in accord with Institutional Review Board procedures at The Icahn School of Medicine at Mount Sinai (ISMMS).

Note that full information on the approval of the study protocol must also be provided in the manuscript.

Field-specific reporting

Please select the one below that is the best fit for your research. If you are not sure, read the appropriate sections before making your selection.

☒ Life sciences ☐ Behavioural & social sciences ☐ Ecological, evolutionary & environmental sciences

For a reference copy of the document with all sections, see nature.com/documents/nr-reporting-summary-flat.pdf

Life sciences study design

All studies must disclose on these points even when the disclosure is negative.

Sample size	Sample sizes were based on experience with the chosen methods used, but guided by power calculations in the cases of the large 'primary' endpoints such as glucose tolerance, body weight.
Data exclusions	There were no data exclusions
Replication	Replication was ensured by using multiple biological replicates, and by using several orthogonal approaches to ensure the reproducibility of our findings..
Randomization	Allocation into groups were based on genotype and the specific animals chosen per group was randomized.
Blinding	All studies that involved manual scoring were conducted in a strictly blinded manner. Other experiments either were split into groups from a single animal or human donor, or were not blinded since the phenotype was so extreme.

Reporting for specific materials, systems and methods

We require information from authors about some types of materials, experimental systems and methods used in many studies. Here, indicate whether each material, system or method listed is relevant to your study. If you are not sure if a list item applies to your research, read the appropriate section before selecting a response.

Materials & experimental systems

n/a	Involved in the study	
☐	☒	Antibodies
☐	☒	Eukaryotic cell lines
☒	☐	Palaeontology and archaeology
☐	☒	Animals and other organisms
☒	☐	Clinical data
☒	☐	Dual use research of concern

Methods

n/a	Involved in the study	
☒	☐	ChIP-seq
☐	☒	Flow cytometry
☒	☐	MRI-based neuroimaging

Antibodies

Antibodies used

Details of antibodies used are in the Methods section and include animal, poly or monoclonal, dilution, company, catalog number, and antibody registration number.

Validation

1. The insulin primary antibody from Dako (Agilent) (A0564) is routinely validated https://www.agilent.com/cs/library/m sds/SDS345 _ NAEnglish.pdf
2. Gucagon Abcam (ab10988). Abcam applies an in-house antibody validation incorporates several advanced technologies a central element of our programme includes the routine use of knockout validation that employs CRISPR gene-edited KO cell lines to provide "true" negative controls. This initiative was recognised with a 2020 CiteAb award for KO validation. https://corporate.abcam.com/ abcam-triumphs-with-industry-award-for-knockout-antibody-validation-success/
3. ChREBP C term (NB400-135) validation from liver extracts form well-fed rats. https://www.novusbio.com/products/chrebp-antibody_nb400-135
4. ChREBP N-term rabbit polyclonal was generated by Genscript and validated in Suppl. Fig. 3 by adenoviral overexpression.
5. anti-Flag rabbit polyclonal from Cell Signaling, where they validate their antibodies in house (https://www.cellsignal.com/about-us/est-antibody-performance-guarantee)
6. Anti-green fluorescent protein (GFP) chicken polyclonal from Aves-Labs (cat# GFP-1020) validated using transgenic mice expressing the GFP gene product (https://www.aveslabs.com/products/anti-green-fluorescent-protein-antibody-gfp)
7. Anti-Cherry (red fluorescent protein) rabbit polyclonal from Rockland ((cat# 600401379S) was validated with purified Red Fluorescent Protein (https://www.rockland.com/categories/primary-antibodies/rfp-antibody-pre-adsorbed-600-401-379/)
8. Anti-Ki67 rabbit monoclonal ThermoScientific (cat# MA5-14520) was validated using serum depleted versus serrum treated HeLa cells (https://www.thermofisher.com/antibody/product/Ki-67-Antibody-clone-SP6-Recombinant-Monoclonal/MA5-14520).

Eukaryotic cell lines

Policy information about cell lines and Sex and Gender in Research

Cell line source(s)

INS1 832/13 cells - Dr. Chris Newgard, Duke University. HEK293 cells were purchased from ATCC (Cat #CRL-1573)

Authentication

Periodic insulin staining and GSIS for INS1 cells. Ability to expand non-replicative adenoviruses for HEK293 cells

Mycoplasma contamination

Both INS1 and HEK293 cells tested negative for mycoplasma.

Commonly misidentified lines (See ICLAC register)

No commonly misidentified lines were used in this study.

Animals and other research organisms

Policy information about studies involving animals; ARRIVE guidelines recommended for reporting animal research, and Sex and Gender in Research

Laboratory animals

Mice were on a C57Bl/6 background, male and female, 6-12 weeks of age. Housing was overseen by the Mount Sinai School of Medicine Center for Comparative Medicine and Surgery in individually-ventilated polysulfone rodent cages, supplied ventilation via a HEPA-filtered supply, wall-mounted unit. Exhaust is connected to the building's exhaust fans with a flexible hose assembly. The temperature and humidity are controlled and there is a 12 hour light and dark cycle.

Wild animals

There were no wild animals used in this study.

Reporting on sex

data was collected primarily on male C57Bl/6 mice, as they are known to be more affected by a high fat diet. However, we also report data on females in the supplemmentray data.

Field-collected samples

No field collected samples were used in this study

Ethics oversight

All protocols were performed with the approval of and in accordance with guidelines established by the Icahn School of Medicine at Mount Sinai Institutional Animal Care and Use Committee.

Note that full information on the approval of the study protocol must also be provided in the manuscript.

Flow Cytometry

Plots

Confirm that:

- [x] The axis labels state the marker and fluorochrome used (e.g. CD4-FITC).
- [x] The axis scales are clearly visible. Include numbers along axes only for bottom left plot of group (a 'group' is an analysis of identical markers).
- [x] All plots are contour plots with outliers or pseudocolor plots.
- [x] A numerical value for number of cells or percentage (with statistics) is provided.

Methodology

Sample preparation	ns-1 cells were co-transfected with px330 containing RNA plasmid and pUC57 containing homology arms and the sequence for mCherry or eGFP. Cells were allowed to recover for 7-10 days following sorting. Sorted populations were then expanded and sorted for additonal 3-5 times until a relatively pure population was obtained
Instrument	BD Influx was used for sorting
Software	BD Spigot
Cell population abundance	The final abundance of cells is marked on Suppl. Fig. 4.
Gating strategy	Debris, dead cells and cell doublets were excluded by side scatter. Non transfected Ins-1 cells were used as a negative control for gating for GFP/mCherry positive cells.

- [x] Tick this box to confirm that a figure exemplifying the gating strategy is provided in the Supplementary Information.
