# Supplementary material for: Maladaptive positive feedback production of ChREBPβ underlies glucotoxic β-cell failure
Source: Nat Commun. 2022 Jul 30;13:4423. doi: 10.1038/s41467-022-32162-x (PMC9339008; doi:10.1038/s41467-022-32162-x)
Supplement: Supplementary file 4 — Description of Additional Supplementary Files [file 41467_2022_32162_MOESM4_ESM.pdf]

Title: Supplementary Movie 1.
Description: Time course of Red/Green INS1 cells after changing media from 2 mM to 20 mM glucose. A field of Yellow (Red/Green) fluorescence in Red/Green INS1 cells from 0 to10 h after changing culture medium from 2 mM to 20 mM glucose.

Title: Supplementary Movie 2.
Description: Pancreata were perfused and isolated from Cre negative male eOEβ mice and clarified using the iDISCO method, and immunolabeled with an antibody against insulin. Lightsheet images were acquired and Imaris software was used to render the video.

Title: Supplementary Movie 3.
Description: Pancreata were perfused and isolated from Cre positive male eOEβ mice and clarified using the iDISCO method, and immunolabeled with an antibody against insulin. Lightsheet images were acquired and Imaris software was used to render the video.
